# Evaluating the effects of archaic protein-altering variants in living human adults

**DOI:** 10.1126/sciadv.ads5703

**Published:** 2025-12-10

**Authors:** Barbara Molz, Mikel Lana Alberro, Else Eising, Dick Schijven, Gökberk Alagöz, Clyde Francks, Simon E. Fisher

**Affiliations:** ^1^ Language and Genetics Department, Max Planck Institute for Psycholinguistics, Nijmegen, 6525 XD, Netherlands.; ^2^ Department of Cognitive Neuroscience, Radboud University Medical Center, Nijmegen, 6525 EN, Netherlands.; ^3^ Donders Institute for Brain, Cognition and Behaviour, Nijmegen, 6525 EN, Netherlands.

## Abstract

Advances in paleogenetics allowed the identification of protein-coding changes unique to *Homo sapiens* by comparing present-day and archaic hominin genomes. So far, experimental validation has been restricted to functional assays and model organisms. Large-scale biobanking now makes it possible to directly assess phenotypic consequences in living adults. Querying exomes of 455,000 UK Biobank participants at 37 sites with supposedly fixed human-specific changes, we identified 103 carriers at 17 positions, with variable allele counts across ancestries. We performed phenotypic evaluations for two example changes. Individuals carrying archaic *SSH2* alleles showed no clear deviations in an array of health, neuropsychiatric, and cognitive traits. Carriers of a *TKTL1* missense variant, previously linked to large effects on cortical neurogenesis, showed no obvious differences in brain anatomy, with many carriers holding college degrees. Our study demonstrates challenges associated with individual interrogation of key sites when seeking insights into the evolution of complex human traits and highlights the importance of including diverse ancestries in biobanking efforts.

## Teaser

Promise and pitfalls of using large biobanks to study impacts of archaic protein-coding variants in living humans.

## INTRODUCTION

Understanding the origins of modern humans and how our ancestors developed sophisticated cultural, social, and behavioral skills has been a central issue for many fields of science (*1*–*3*). Although latest research is gradually reaching a consensus that cognitive capacities of Neanderthals were greater than previously appreciated, the question remains why *Homo sapiens* outlived its archaic cousins and was able to migrate all across the globe (*3*–*6*). Advances in high-throughput DNA sequencing and the availability of three high-quality Neanderthal genomes (*7*–*9*) enabled comparative genomic approaches, opening up ways to reconstruct aspects of the evolutionary history of *H. sapiens*. In particular, such approaches yielded catalogs of missense variants (changes that substitute one amino acid for another in an encoded protein) that occurred after *H. sapiens* split from its common ancestor with Neanderthals ~600,000 years ago, and that reached (near) fixation on our lineage. These human-specific fixed derived alleles have been hailed as promising entry points for explaining human origins, given their enrichment in genes that are relevant for human-specific traits and involved in cortical development and neurogenesis (*1*, *3*, *8*).

Because a missense variant can potentially arise and spread through a population without any consequence to properties or functions of the encoded protein, experimental validation is crucial to determine the functional impacts of derived alleles. In a prominent example, Pinson *et al.* (*10*) investigated the impacts of a lysine-to-arginine substitution in human *TKTL1* (chrX:154315258; G>A) by comparing the archaic and derived alleles using genome-edited cerebral organoid and in vivo models, as well as in primary brain tissue. The authors observed substantial differences between samples carrying the Neanderthal and *H. sapiens* versions of *TKTL1* in basal radial glia abundance and neurogenesis, and suggested that the modern human-derived allele might have played a key role in evolutionary expansion of the brain’s frontal lobe. However, despite the multiple strengths of cerebral organoids for modeling events in early embryogenesis (*11*), cellular diversity and transcriptomic programs of these models do not fully recapture human brain development, and lack insights from diversity across genetic ancestries (*12*). Similarly, expression of “humanized” genes in primary brain tissue of nonhuman species may lead to nonspecific artefacts (*12*–*14*), due to interspecies differences in genetic background. Thus, the actual consequences of any such modern human-derived genetic changes may be more complex than those that can be observed in cellular/animal models (*15*).

A complementary approach for evaluating broader biological impact, one that has only recently become feasible, depends on the identification of present-day living carriers of archaic alleles at genomic positions that differ between modern humans and Neanderthals (*2*). Databases like gnomAD highlight the existence of individuals carrying these archaic single nucleotide variants (aSNVs) (*12*), albeit in low numbers. With availability of large-scale biobanks with exome sequencing and trait data, it is now possible not only to detect aSNVs in living humans, but also to investigate putative phenotypic consequences in a way that could not be done before.

In this study, we used the UK Biobank (UKB), a large-scale population resource with both exome and dense phenotype data available from around half a million individuals (*16*). This offers a unique opportunity to (i) determine the frequencies of present-day aSNV carriers and (ii) assess how phenotypic profiles of carriers of the archaic allele compare to individuals that are homozygous for the derived present-day human allele. We focused our efforts on a catalog of putative fixed genomic positions established from a prior survey of potential human-specific changes (*3*) and searched for carriers of ancestral alleles among UKB participants. To gain insight into the phenotypic profile of an exemplary aSNV in *SSH2*, we contrasted identified carriers with a curated set of noncarriers, homozygous for the derived allele, assessing a range of traits. Given the especially dramatic effects of the *TKTL1* aSNV on neurogenesis reported by Pinson *et al.* (*10*) in their cellular and animal models, we also included this high-frequency human-specific change in our investigations. Specifically, we identified carriers of the archaic *TKTL1* allele and used the available neuroimaging data (*17*) to study putative effects of the aSNV on brain morphology and cognitive traits. We use our findings to make recommendations about how to optimize future biobank-based investigations of human evolution.

## RESULTS

### A total of 103 carriers of aSNVs in 17 positions are identified in the UKB across different ancestries

In the first part of the study, based on the Kuhlwilm and Boeckx (*3*) catalog of single-nucleotide changes that distinguish modern humans and archaic hominins, we curated a list of 42 fixed missense changes with an allele frequency of one (AF = 1) at the time of publication, indicating complete fixation within the investigated modern human populations (see Materials and Methods and table S1). After quality control (QC; see Materials and Methods), we then queried the whole-exome sequencing data of ~455,000 individuals (*18*–*20*) of the UKB to identify possible carriers of the archaic allele at 37 positions. We investigated four ancestry superclusters: labeled as European, African, East, and South Asian (fig. S1A). In total, we identified 103 unique individuals carrying 118 aSNVs in 13 protein-coding genes (Table 1). All were heterozygous carriers, except for a female carrying a homozygous aSNV in *GRM6* (chr5:178994530), a gene encoding the ON bipolar metabotropic glutamate receptor, which overall also represents the genomic position with the largest carrier count.

**Table 1.** Overview of identified aSNV carriers in the UKB. Genotype count of carriers of each aSNV and respective individuals homozygous for the derived allele are noted per ancestry supercluster. Genomic positions are based on hg38. Het, heterozygous; Hom, homozygous; Ref, reference allele; SAS, South Asian; EAS, East Asian; EUR, European; AFR, African.

Gene	*KIF26B*		*NOTO*		*GRM6*			*ADAM18*		*ADAM18*		*DCHS1*		*KNL1*		*KNL1*		
Chromosome	1		2		5			8		8		11		15		15		
Position (hg 38)	245419603		73210883		178994530			39680099		39706833		6633538		40620662		40623442		
Reference allele	A		T		G			C		G		C		G		A		
Archaic allele	G		A		T			T		A		T		A		G		
	**Het**	**Hom**	**Het**	**Hom**	**Hom**	**Het**	**Hom**	**Het**	**Hom**	**Het**	**Hom**	**Het**	**Hom**	**Het**	**Hom**	**Het**	**Hom**	
		**(Ref)**		**(Ref)**	**(Alt)**		**(Ref)**		**(Ref)**		**(Ref)**		**(Ref)**		**(Ref)**		**(Ref)**	
**SAS**		8585		8585			8585		8585		8570		8585		8583		8585	
**EAS**		1887	3	1884			1886	2	1885	2	1881		1887	1	1885	1	1886	
**EUR**		423885		423883		3	423838	1	423882		423457		423887	3	423652		423885	
**AFR**	2	5090		5092		12	5077		5092		5075		5092		5087		5092	
**Uncategorized**	3	13343	3	13343	1	28	13316		13346		13319	1	13345	2	13339	1	13345	
**Total *N***	**5**	**452790**	**6**	**452787**	**1**	**43**	**452702**	**3**	**452790**	**2**	**452302**	**1**	**452796**	**6**	**452546**	**2**	**452793**	
																		
**Gene**	***ZNF106***		***SPAG5***		***SPAG5***		***SPAG5***		***SSH2***		***RFNG***		***GREB1L***		***LMNB2***		***C3***	
Chromosome	15		17		17		17		17		17		18		19		19	
Position (hg 38)	42450114		28592016		28592759		28598560		29632016		82049104		21505418		2434035		6685100	
Reference allele	C		G		C		A		T		G		A		A		G	
Archaic allele	T		C		T		G		C		A		G		T		A	
	**Het**	**Hom**	**Het**	**Hom**	**Het**	**Hom**	**Het**	**Hom**	**Het**	**Hom**	**Het**	**Hom**	**Het**	**Hom**	**Het**	**Hom**	**Het**	**Hom**
		**(Ref)**		**(Ref)**		**(Ref)**		**(Ref)**		**(Ref)**		**(Ref)**		**(Ref)**		**(Ref)**		**(Ref)**
**SAS**		8585		8585		8584		8585		8585		8584		8585		8585		8584
**EAS**	4	1883		1887		1887		1887		1887		1887	1	1886	2	1885		1887
**EUR**		423887		423887	1	423883		423885	21	423866	2	423832		423877		423886	2	423861
**AFR**		5092	5	5087	5	5087	5	5087		5092		5092		5092		5092		5092
**Uncategorized**	1	13345		13346		13346		13346		13345		13343		13346		13346		13346
**Total *N***	**5**	**452792**	**5**	**452792**	**6**	**452787**	**5**	**452790**	**21**	**452775**	**2**	**452738**	**1**	**452786**	**2**	**452794**	**2**	**452770**

We observed diverging carrier counts for aSNVs across ancestry superclusters. Even though the UKB is composed of predominantly European ancestry individuals (*16*) with only around 0.5% individuals of African and 1% of East Asian descent, nearly equal numbers of aSNV carriers were identified in the European, East Asian, and African ancestry superclusters, highlighting allele frequency differences for these rare variants, and a bias toward European data being used to originally identify aSNVs.

We identified five individuals carrying a combination of three aSNVs in *SPAG5* (chr17:28592016; chr17:28592759; and chr17:28598560), and two pairs of carriers who carry a combination of two aSNVs in *ADAM18* and *KNL1*, respectively (*ADAM18*: chr8:39680099 and chr8:39706833; *KNL1*: chr15:40620662 and chr15:40623442). In each case, the aSNVs found in the same carriers were in tight linkage disequilibrium and thus were likely inherited together.

We also queried the relatedness status (up to third degree) of identified carriers and found only one related pair carrying an aSNV, for a variant in *SSH2*. Thus, it is unlikely that the allele counts of identified aSNVs in our study are inflated due to relatedness.

### Phenotypes assessed in carriers of the *SSH2* aSNV do not deviate from matched noncarriers

In the second part of the study, we considered how identification of aSNV carriers with phenotypic information in the UKB could be used to gain further insights into potential biological effects. The small sample sizes of aSNV carriers coupled to the complexity of the data (including information available across many traits, as well as differing allele frequencies depending on ancestral background) hinder formal statistical analyses; for example, systematic unconstrained phenome-wide association screens are clearly underpowered in this context. Nonetheless, as outlined above, prior literature has postulated protein-coding aSNVs as key drivers of the emergence of distinctive traits of modern humans, relying on assumptions of moderate-to-strong effect sizes (*1*–*3*, *7*). If such claims are valid, we might reasonably expect relevant phenotypes in carriers of aSNVs to fall outside (or at the extremes of) the typical range observed in control individuals in a way that may be apparent from qualitative comparisons. When making such comparisons, careful curation of matched controls, ensuring cases and controls are aligned in terms of age, sex, and ancestry, can minimize impact of confounding factors and maximize informativeness.

We selected an aSNV in *SSH2* (chr17:29632016) to exemplify this strategy, because the encoded protein is a protein phosphatase with enzymatic properties regulating actin filament dynamics and possible functions in neurite outgrowth (*3*, *21*–*23*), suggesting that variations could potentially affect cellular processes. Furthermore, the variant was found in a relatively large number of unrelated carriers within a strict ancestry cluster (*N* = 19; see Materials and Methods), a sample size that would have sufficient power to support detection of putative large rare-variant effects (β > 0.5766) with high confidence (Materials and Methods and fig. S4). To limit the phenotypic search space, we chose the following traits: body composition measures [body mass index (BMI) and whole body fat mass], height, overall health rating, smoking status, and highest qualification level as an indication of educational attainment. These phenotypes were selected a priori based on previously identified Genome-Wide Association Study (GWAS) trait associations of *SSH2* (*24*) that further overlapped with traits linked to Neanderthal admixture (*1*, *25*–*29*).

**Fig. 1.**
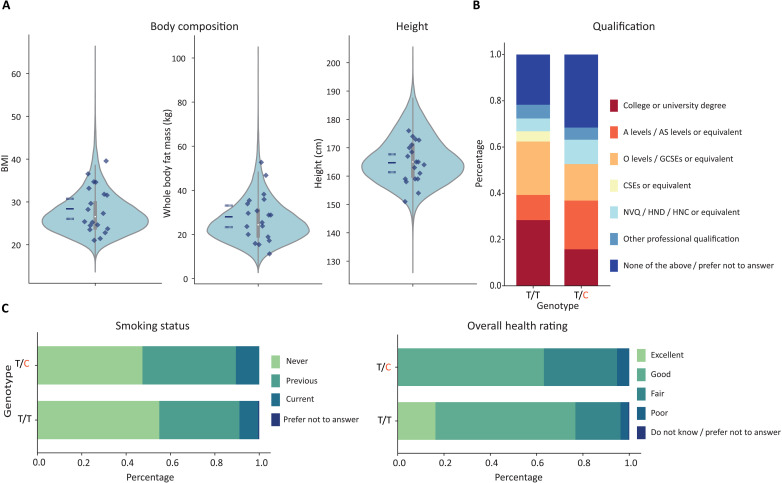
Investigating phenotypic effects in carriers of the *SSH2* aSNV. (**A**) Values of continuous traits are shown for each aSNV carrier as dark blue diamonds. Violin plots show the phenotypic distribution of matched set of noncarriers, with boxplots indicating the 25th and 75th percentiles, and whiskers representing 1.5 times the interquartile range (IQR); mean and 95% confidence interval of the aSNV carriers are indicated as blue solid and dotted lines, respectively. (**B** and **C**) Stacked bar plots showing the percentage of highest qualification level, as well as health-related measures for each genotype: T/T for matched noncarriers and T/C for aSNV carriers (*N*_aSNV_ = 19; *N*_Noncarrier_ = 39,501). A level = Advanced level, AS level = Advanced Subsidiary level, O level = Ordinary level, GCSE = General Certificate of Secondary Education, CSE = Certificate of Secondary Education, NVQ = National Vocational Qualification, HND = Higher National Diploma, HNC = Higher National Certificate. See table S1 for categorical trait percentages with 95% binomial confidence intervals.

For all continuous traits, carriers of the *SSH2* aSNV were within the standard trait distribution based on a matched set of individuals homozygous for the derived alleles (noncarriers; see Materials and Methods) and were evenly distributed throughout the full sample range (Fig. 1A). A similar pattern was observed for categorical traits, where carriers show no strong deviating pattern from the matched noncarrier cohort (Fig. 1, B and C). Although the confidence intervals (table S1) are broad for aSNV carriers, the pattern of findings appears inconsistent with existence of large or extreme effect sizes, in line with the conclusion that any potential effects, should they exist, are likely modest. Given its putative roles in neurite outgrowth, prior associations of common variants with a broad array of brain imaging metrics (*24*), and the general involvement of protein phosphatases in psychiatric and neurological disorders (*30*), we investigated the possible consequences of carrying aSNVs in *SSH2* for a range of neuropsychiatric traits. We did not observe diverging patterns for aSNV carriers compared to the noncarrier group (fig. S2 and table S2).

### The archaic allele in *TKTL1* shows little consequence for frontal lobe morphology and overall cognition in adult humans

As illustrated by the above example, the limited prior work on aSNVs can make it difficult to specify clear hypotheses over which phenotypic traits would be most expected to show signs of functional effects. Therefore, we next investigated a variant for which previous research yields strong specific predictions over likely impacts on brain and cognition: the archaic allele (A) of the rs111811311 polymorphism (A/G) of the *TKTL1* gene, located on the X chromosome. This missense change (yielding a lysine-to-arginine change at residue 317 of the long isoform) gained considerable prominence in recent literature when it was proposed by Pinson *et al.* (*10*) as a major driver of human/Neanderthal brain differences in evolution based on an array of functional experiments. The variant was not among the curated list of aSNVs described in the first part of our study, because it did not fit the criteria of full fixation in the analyses performed by Kuhlwilm and Boeckx (*3*) (AF = 1). Moreover, a comment in response to Pinson *et al.* (*10*) has highlighted the existence of rs111811311 archaic allele carriers in gnomAD (*12*), but without any phenotypic follow-up.

Querying the UKB resource for the *TKTL1* archaic allele, we identified 45 heterozygous and one homozygous female carrier, as well as 16 hemizygous male carriers across multiple ancestry groups (Table 2 and fig. S1C). Among these 62 carriers, we identified four pairs with a kinship coefficient below 0.042, indicating again that relatedness (up to the third degree) is unlikely to explain the larger number of carriers. One individual was identified with an archaic allele in both *TKTL1* and *KIF26B*.

**Table 2.** Overview of identified aSNV carriers for *TKTL1* in the UKB. Genotype count of carriers of the archaic allele and individuals homozygous or hemizygous for the derived allele are noted per ancestry supercluster. Genomic positions are based on hg38. Alt, alternative/ancestral allele; Ref, reference allele; rsID, reference SNP-cluster identification.

*TKTL1*		Population	Homozygotes (Alt)	Hemizygotes (Alt)	Heterozygotes	Hemizygotes (Ref)	Homozygotes (Ref)
Chromosome	X	SAS				4612	3960
Position (hg38)	154315258	EAS				600	1283
Reference allele	G	EUR		4	12	193302	229987
Archaic allele	A	AFR		1	10	2251	2822
rsID	rs111811311	Uncategorized	1	11	23	5774	7513
		**Total *N***	**1**	**16**	**45**	**206539**	**245565**

**Fig. 2.**
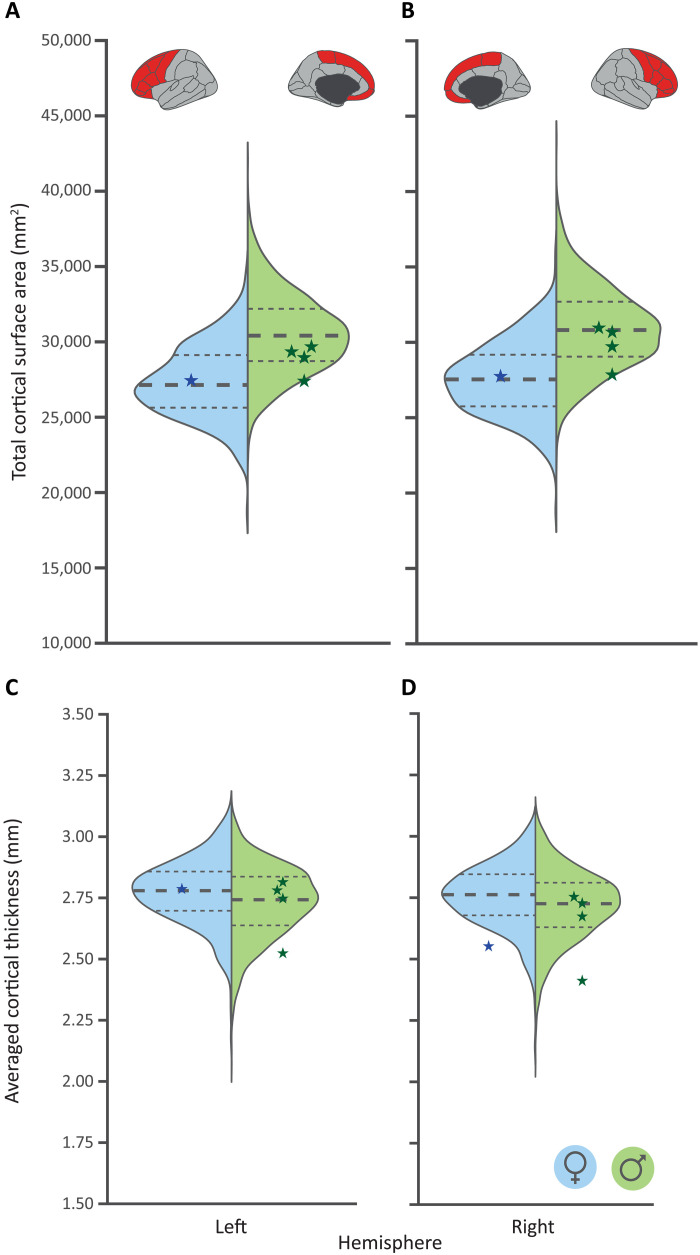
Carriers of the archaic allele of the *TKTL1* aSNV show no diverging cortical measures compared to a matched set of noncarriers. Archaic allele carrier values for each sex and metric are depicted as diamonds. These are overlaid over split violin plots indicating the phenotypic variability for both the female (left, blue) and male (right, green) matched noncarrier sample for total frontal lobe cortical surface area for both left and right hemisphere (**A** and **B**, respectively) and averaged frontal lobe cortical thickness (**C** and **D**, respectively). (*N*_aSNV_ = 5; *N*_Noncarrier_ = 2145.) Thick dotted line indicates the median of the noncarrier sample, while the thin dotted lines highlight the 25th and 75th percentiles.

Given that the cellular/animal work of Pinson *et al.* (*10*) linked the human-derived allele of *TKTL1* to substantial increases in neuron production in the prefrontal cortex, we contrasted imaging-derived structural brain metrics of the frontal lobe in unrelated aSNV carriers (*N* = 5) and matched noncarriers, homozygous for the derived allele (*N* = 2145) to investigate the effects of carrying an archaic allele on frontal lobe surface area and cortical thickness in living human adults (Fig. 2). We found that the range of phenotypic variation of aSNV carriers lies mainly within the 25th and 75th percentiles of the noncarriers for all cortical measures. This finding is in contrast to the pronounced effects shown in the various investigations performed by Pinson *et al.* (*10*), which would predict substantial reductions in prefrontal cortex brain metrics of carriers of the archaic allele. As a sensitivity analysis, we repeated this approach in an ancestry matched cohort of only European carriers (*N* = 3) and a matched noncarrier cohort (*N* = 30) and obtained an even clearer overlap in phenotypic distributions (fig. S3).

**Fig. 3.**
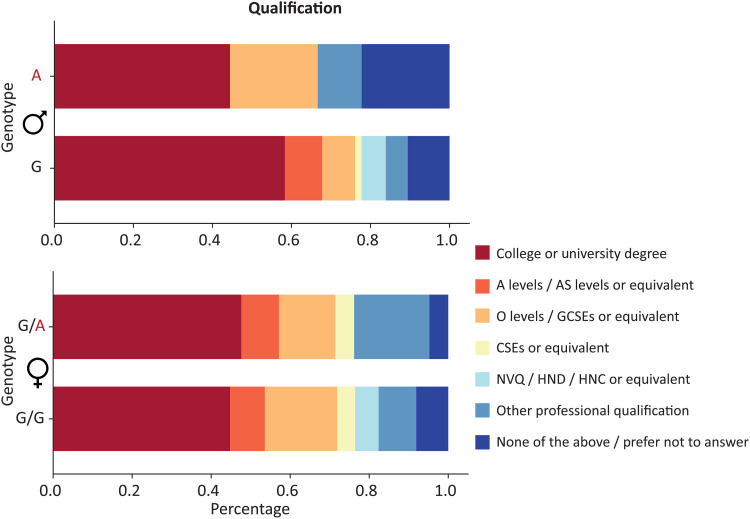
Qualification levels of carriers of archaic alleles of the *TKTL1* aSNV are similar to a matched set of noncarriers. Stacked bar plots showing the percentage of highest qualification level by genotype (male sample: *N*_aSNV_ = 9, *N*_Noncarriers_ = 180; female sample: *N*_aSNV_ = 21, *N*_Noncarriers_ = 420). G, reference/derived allele; A, archaic allele. A level = Advanced level, AS level = Advanced Subsidiary level, O level = Ordinary level, GCSE = General Certificate of Secondary Education, CSE = Certificate of Secondary Education, NVQ = National Vocational Qualification, HND = Higher National Diploma, HNC = Higher National Certificate. See table S3 for trait percentages with 95% binomial confidence intervals.

Increased neuronal proliferation and expansion of the neocortex along the lineage leading to modern humans are argued by some to have been a driver of increased cognitive capacities of our species (*31*, *32*). On the basis of the findings of Pinson *et al.* (*10*), some other researchers and commentators have proposed that the *TKTL1* protein-coding aSNV contributed to differences in cognition between *H. sapiens* and extinct archaic humans (*33*). Thus, we also assessed educational qualification levels of carriers of the archaic *TKTL1* allele (*N* = 30) compared to matched noncarriers (*N* = 600) (Fig. 3 and table S3). Due to the difference in zygosity, this was done separately for males and females.

While the percentage of males with the highest qualification level was slightly lower for those with the archaic allele of *TKTL1*, it is notable that, in both sexes, a substantial proportion of carriers of this allele have a college or university degree. In particular, four of the nine males with only an archaic allele on this polymorphic site of the X chromosome have a college/university degree. Despite the wide confidence interval, we do not observe the kind of extreme reduction in higher qualifications in aSNV carriers that would be consistent with a major cognitive effect. Thus, the pattern of findings casts doubt on the idea that the human-derived change in *TKTL1* was central to the evolution of enhanced human cognitive abilities (*33*).

## DISCUSSION

This study brings an innovative source of empirical data to questions regarding evolutionary impacts of protein-coding variants that distinguish between modern humans and our extinct archaic cousins, adding to the rich prior literature in this area, recently reviewed by Zeberg and colleagues (*2*). Our work identified 165 unique carriers of aSNVs for 18 out of a total of 38 interrogated genomic positions in around 450,000 individuals with exome data in the UKB. Regarding phenotypic consequences of an exemplar aSNV in *SSH2*, one for which relatively large numbers of carriers were available, all interrogated traits fell within the typical range of variation, with no obvious divergence from the norm. A similar pattern was observable for *TKTL1* for frontal lobe structural measures as well as overall qualification level, despite this variant previously showing large effects on neocortical development in cellular/animal models.

Ever since the first high-coverage genome sequence of a Neanderthal resulted in a catalog of fixed missense aSNVs (*7*), the overall number has continually decreased, as more high-quality Neanderthal genomes and ever-increasing population databases of present-day humans have become available. For such protein-coding changes, the present study reduces the number of potential fully fixed genomic positions described in Kuhlwilm and Boeckx (*3*) that we investigated here from 37 to only 20, while the true number is likely even smaller. This raises questions over whether (some of) the aSNV carriers are explained by rare back-mutations, whether these sites were never fixed to begin with, or whether the ancestral allele was reintroduced postfixation during admixture events (*2*). Whereas for some genomic positions, only a handful of carriers were found in the UKB, some positions present with higher carrier counts that make back-mutations an unlikely explanation (*1*). Furthermore, higher ancestral allele counts were often evident in non-European ancestry groups. High genetic diversity within African populations (*34*) might partially explain this pattern, but considering the skewness of the UKB toward White European ancestry (*16*), it remains intriguing. Although it is known that some isolated populations have higher levels of archaic ancestry, either because they persisted since a common ancestor, as seen in the Khoe-San (*35*) who retained some ancestral human variation, or due to relatively recent admixture with Neanderthals/Denisovans (e.g., Oceanian populations) (*36*, *37*), there is no detailed catalog of fixed human-specific changes across a range of ancestries that could be used as a reference point, given that most results of genomic studies are solely based on populations with European ancestry (*2*).

The presence of aSNV carriers in population databases, however, does not itself rule out the possibility that these DNA changes contributed to the emergence of anatomically modern humans. While experimental validation in model systems is crucial to understand the impact of variation at these genomic positions, current approaches are laborious, with a range of known pitfalls (*12*, *38*). The availability of exomic and phenotypic data in a resource like the UKB makes it possible for the first time to query possible phenotypic consequences in present-day adult living humans that carry the variants of interest.

The aSNV on *SSH2* was carefully chosen as a best-case example based on its putative impacts on enzymatic properties of the encoded protein, suggesting a potentially large effect size at the cellular level, and on the relatively large number of identified carriers thus allowing sufficient power to observe profound phenotypic consequences. None of the investigated traits of interest here indicated unambiguous deviations between matched individuals homozygous for the derived allele and aSNV carriers, suggesting that there are no extreme phenotypic consequences at the tails of the distribution of carrying an ancestral rather than a derived version at the queried position. When alleles are in the heterozygous state, this may mask phenotypic consequences, perhaps leading to subtle effects that would be difficult to detect with our available sample sizes. In general, the absence of a clear pattern here does not necessarily indicate the absence of an effect altogether; rather, it is possible that the true effect size is much smaller than anticipated and would require a substantially larger sample to be detectable. Moreover, lacking power for a systematic phenome-wide screen, we chose phenotypes to target a priori based on broader literature and might have thus missed a trait that is truly affected by the variant. This raises a larger question of importance for the field: which phenotype(s) would best represent “the human condition” in investigations of this kind? Latest archaeological evidence increasingly suggests cognitive and behavioral similarities with our extinct archaic cousins, meaning that differences, especially for complex traits, may well be subtle (*3*–*6*). The lower carrier numbers of other aSNVs (at least within ancestry clusters) further limit the scope of currently feasible phenotypic investigations. A large phenome-wide scan sensitive enough to detect small deviations from the norm might highlight the most important phenotypes, as well as clarify contributions of these genomic positions, but this will only be tractable when even larger sample sizes are available than at present.

While Kuhlwilm and Boeckx (*3*) already showed that the aSNV in *TKTL1* is not fixed in modern populations but is instead a human-specific high-frequency variant, several reasons led us to include this variant in the second stage of the current investigation. First, effects on brain development of ancestral versus derived alleles of this aSNV are well described in Pinson *et al.* (*10*), based on their in-depth experiments in animal/cellular models, allowing for a more targeted phenotypic selection. Complementing findings from these models, the researchers also reported that disrupting *TKTL1* expression in fetal human brain neocortical tissue significantly reduced basal radial glial progenitors (*10*). Second, the effect sizes of the aSNV allele reported in Pinson *et al.* (*10*) were substantial, indicating that, even with only a small number of identified carriers, there should be good prospects of detecting such phenotypic consequences. Third, the position of the aSNV on the X chromosome should lead to even more pronounced effects in males, who are hemizygous for either a derived or ancestral allele. Still, we did not see large systematic deviations at the extremes of the phenotypic distribution in neuroanatomical properties of the frontal lobe even in male carriers. In addition, a substantial proportion of these had a college/university degree, also arguing against a major impact on cognitive function. While the absence of consequences for adults might possibly be explained by compensatory mechanisms with mitigating effects on the developing frontal lobes, our results show that effect sizes identified in functional assays and model organisms cannot be directly extrapolated to the consequences of carrying these changes for adult human phenotypes (*15*).

For the second part of the study, even though carrier sample sizes are small, they should still have sufficient power to detect strong, consistent phenotypic deviations of the kind expected under the large-effect rare-variant model assumed by prior aSNV literature (see Materials and Methods). While large confidence intervals were observed (tables S1 to S3), reflecting limitations in precision for the available sample size, an absence of major deviations across the investigated traits suggest that no extreme effects are present. We do not draw inferences here about the potential existence of more modest effect sizes, given lack of power to detect those (fig. S4). We would caution against attempting to draw conclusions from observing more subtle shifts in trait patterns compared to matched controls. With the limited numbers of carriers available to analyze, the low power for detecting true differences of smaller effect size is accompanied by an elevated false-positive rate and a risk of overinterpreting noise (*39*). A much larger sample size would be necessary to determine whether comparable results or subtle differences in profiles of *SSH2* and *TKTL1* aSNV carriers represent meaningful results or are simply random variations.

Beyond general challenges related to rare variant analysis and the choice of target phenotypes, as discussed above, limitations of the current study include those related to the nature of the UKB cohort [restricted age range, lack of diversity in ancestral background, and existence of participation bias (*16*, *40*)] and the need for more and better-quality archaic hominin genomes to understand the genetic variation patterns in their populations. Moreover, large-scale biobanking cohorts like the UKB seldom collect information on some of the most pertinent traits for understanding human origins; they lack measures or assessments of speech and language abilities (*41*), which further restricts the range of evolutionary questions they can address (*42*). A recent investigation of a human-specific high-frequency protein-coding change in *NOVA1* [excluded from the Kuhlwilm and Boeckx (*3*) catalog due to its lack of complete fixation] suggested this variant to be a major driver of language evolution, based primarily on observations of slight alterations in properties of vocal calls of a knock-in mouse (*43*). Without information on the speech and language abilities of human carriers of this same variant, links to spoken language capacities must remain speculative.

The findings of our study resonate well with the recent perspective of the field set out by Zeberg and colleagues (*2*). With this concrete demonstration of biobank analyses, we provide impetus toward promising avenues for future investigations: (i) The inclusion of more large-scale diverse population databases (*44*–*47*) together with the information from the third high-quality Neanderthal genome (*9*) (and additional archaic genomes that might be sequenced) will likely yield a more representative catalog of human-specific changes to help reconstruct how natural selection, archaic gene flow, and our demographic history together shaped our genome (*1*, *2*, *34*, *48*). (ii) Given the ever-decreasing number of such sites, it seems warranted to abandon the notion of fully fixed variants, broadening the scope to also take high-frequency nonfixed changes into account. Kuhlwilm and Boeckx (*3*) already made a start in this direction, but with the availability of larger and more diverse databases, this broader list will need updating. As more population databases are also including whole genome sequencing, the search can be expanded further to include high-frequency changes in regulatory regions (*49*). (iii) Our results indicate that looking at each of these genomic positions individually might not be so informative and that future work focusing on their aggregated effects could be valuable (*2*, *3*). One way to achieve this would be by grouping high-frequency changes according to their potential functions [see Kuhlwilm and Boeckx (*3*) for an initial categorization]. Furthermore, a list of high-frequency variants could also be used for burden testing, which would additionally allow formal statistical analyses of possible effects (*50*, *51*). (iv) Recent comparative genomic studies of primates have uncovered variants that were previously thought to be unique to modern humans (*52*). Incorporating also these kinds of comparative perspectives could provide further clarity over the functional relevance of aSNVs, as genetic changes at sites that are not highly conserved within and across primate species are less likely to have major phenotypic consequences in humans.

Overall, by leveraging the availability of archaic variation in modern biobanks, our study has provided evidence against the notion of fixed genomic changes on the human lineage, highlighted the challenges associated with individual interrogation of key sites when seeking insights into the emergence of complex human traits, and emphasizes again the importance of including diverse ancestral backgrounds in studies on the origins of our species.

## MATERIALS AND METHODS

### Dataset

All data used were obtained from the UKB under the research application 16066 with C. Francks as the principal investigator. Detailed descriptions of the data used as well as sample, genotype, and variant-specific QC are given below. The UKB has received ethical approval from the National Research Ethics Service Committee North West–Haydock (reference 11/NW/0382), and all of their procedures were performed in accordance with the World Medical Association. Informed consent was obtained for all participants by the UKB with details about data collection and ethical procedures described elsewhere (*16*, *53*).

### Whole-exome sequencing data

Whole-exome sequencing was performed, and data were processed by the UKB according to protocols described elsewhere (*18*–*20*). Briefly, the samples were multiplexed and then sequenced using 75–base pair paired-end reads with two 10–base pair index reads on the Illumina NovaSeq 6000 platform using either S2 (first exome release) or S4 flow cells. Sample-specific FASTQ files, representing all the reads generated for that sample, were mapped to the GRCh38 genome reference with BWA-MEM (*54*). Subsequently, the binary alignment files (BAM) for each sample contained the mapped reads’ genomic coordinates, quality information, and the degree to which a particular read differed from the reference at its mapped location. Duplicated reads were removed with the Picard (*55*) MarkDuplicates tool. Genomic Variant Call Format (GVCF) files were then produced using the weCall variant caller. Upon completion of variant calling, individual sample BAM files were converted to fully lossless Compressed Reference-oriented Alignment Map (CRAM) files using SAMtools (*56*).

For this project, we made use of the Broad 455k exome gnomAD VCF files (UKB data field 24068): population VCF files that have been returned to the UKB as part of the “alternative exome processing” (UKB category 172). Here, original UKB CRAM files were reprocessed according to the Genome Analysis Toolkit (GATK) Best Practices, aligning reads using BWA-MEM 0.7.15.r1140 and processing reads using Picard and GATK with protocols described in detail in Karczewski *et al.* (*57*).

### Variant selection

We based our selection of fixed, amino-acid changing SNVs on a list of high-frequency human-specific missense changes described in Kuhlwilm and Boeckx (*3*), where only those SNVs with an allele frequency of one were selected, indicating total fixation at the time of their publication. These 42 genomic positions were lifted to GRCh38 using Liftover (https://liftover.broadinstitute.org/). We further confirmed that each SNV was located in a translated exon using gnomAD v3.1.2 (https://gnomad.broadinstitute.org/) and the UCSC Genome Browser (https://genome.ucsc.edu/), which led to exclusion of three variants located in *C1orf159* (chr1:1091245), *DNHD1* (chr11:6534188), and *DNMT3L* (chr21:44251169). One genomic position in *TBC1D3* (chr17:38202786) was excluded due to ambiguous results after liftover to GRCh38.

Given its reported profound effects on brain development in cellular/animal models (*10*), an SNV in *TKTL1* on chromosome X (chrX:154315258), previously reported as fixed by Prüfer *et al.* (*7*), was also included in our analysis despite being reported as a high-frequency variant (AF: 0.999694) in Kuhlwilm and Boeckx (*3*). This resulted in a total of 39 SNVs in 33 genes that were put forward for further analysis (see table S1).

### Sample specific QC

For all available individuals included in the gnomAD VCFs (*N* = 454,672), we first applied sample-level QC measures. This entailed excluding individuals with a mismatch of their self-reported (UKB data field 31) and genetically inferred sex (UKB data field 22001), as well as individuals with putative aneuploidies (UKB data field 22019), or individuals who were determined as outliers based on heterozygosity [principal components (PC)–corrected heterozygosity > 0.1903] or genotype missingness rate (missing rate > 0.05) (UKB data field 22027), leading to a final sample of 452,797 individuals.

### Variant and genotype QC

For further analysis, we moved to the UK Biobank Research Analysis Platform (UKB RAP; https://ukbiobank.dnanexus.com) and queried the curated list of genomic position detailed above using bcftools (version 1.17) (*58*) to identify possible carriers of the archaic allele. To assure that identified carriers did not represent sequencing errors, we only included aSNVs that were called with PASS in the VCF. This variant filter is based on a combination of a random forest classifier and hard filters, detailed in Karczewski *et al.* (*57*). Only one SNV (chr9:6606647) did not pass these filters and was discarded for further analysis. We further used Hail (https://github.com/hail-is/hail) as implemented in JupyterLab on the UKB RAP for QC of individual genotype data, where genotypes at the specific positions were filtered based on genotype quality (QUAL > 20), depth (DP > 10), and allele balance for heterozygous genotypes (AB > 25% < 75%), leading to different sample counts per queried position.

### Variant distribution per ancestry cluster

We inferred ancestry for all individuals passing the QC outlined above. We first used the self-reported ancestral background (*16*) (UKB data field 21000-0.0) as provided by the UKB and grouped each individual into four major ancestry clusters [European (EUR), African (AFR), South Asian (SAS), and East Asian (EAS)]. Individuals who reported “Mixed,” “Other,” “Do not know,” and “Do not want to answer” were grouped as “Uncategorized.” We then used the first four provided genetic ancestry PCs (UKB data field 22009-0.1 to 22009-0.4) and assigned each individual to one of the respective major ancestry clusters using hard cutoffs. Individuals labeled as “Uncategorized,” which only showed a highly dispersed cluster, were reassigned to one of four superclusters if their PCs fell within the respective boundaries. Otherwise, the individuals kept their initial category. This resulted in 5092 individuals within the AFR ancestry cluster, 1887 individuals within the EAS cluster, 423,887 individuals within the EUR ancestry cluster, and 8585 individuals within the SAS cluster, whereas 13,346 individuals could not be assigned to one of the superclusters and were thus labeled as “Uncategorized.”

### Relatedness

To infer whether relatedness could explain an accumulation of aSNVs at certain positions, we identified whether carriers had a kinship coefficient > 0.0442 (UKB data field 22021) but initially did not exclude any individuals based on relatedness. For phenotypic analysis, this information was used, and one individual from each pair of relatives was excluded, where we prioritized the exclusion of noncarriers, as well as individuals related to a larger number of other individuals.

### Phenotypic analyses

#### SSH2

For phenotypic analysis, we chose one exemplary genomic position from our queried list for a qualitative comparison of carriers to noncarriers. Our initial query of the exome data highlighted a group of 21 individuals with an aSNV on chr17:29632016 within *SSH2*, where all 21 were grouped within the EUR ancestry cluster, and 20 were also assigned to the more stringent “White British” ancestry (UKB data field 22006) (*16*). We further excluded one carrier due to relatedness, leading to a final carrier count of 19 unrelated, White British individuals (14 females; mean age ± SD: 59.42 ± 7.94 years).

We derived an age- (UKB data field 21003-0.0), sex- (UKB data field 31-0.0), and ancestry-matched (White British, UKB data field 22006) unrelated sample of individuals homozygous for the derived allele, where each carrier was paired with 2079 unique noncarriers. This led to a matched noncarrier cohort of 39,501 individuals (29,106 females; mean age ± SD: 60.69 ± 7.73 years).

#### Trait selection

Based on literature detailing the phenotypic legacy of previous admixture events and previous genetic associations with *SSH2* variants as detailed in the GWAS catalog, we selected a range of traits to investigate potential phenotypic effects of carrying an aSNV: BMI (UKB data field 21001-0.0), whole-body-fat-mass (UKB data field 23100-0.0), height (UKB data filed 50-0.0), overall health rating (UKB data field 2178-0.0), smoking status (UKB data field 20116-0.0), and qualification (UKB data field 6138-0.0). For each individual, we included only the highest qualification level reported in the analysis. As the GWAS catalog highlighted several associations of *SSH2* with different brain-related phenotypes and given prior proposed links of archaic admixture to psychiatric disorders, we also included a range of neuropsychiatric metrics: “Seen doctor (GP) for nerves, anxiety, tension or depression” (UKB data field 2190-0.0), “Seen psychiatrist for nerves, anxiety, tension, or depression” (UKB data field 2100-0.0), moodiness (UKB data field 1920-0.0), miserableness (UKB data field 1930-0.0), loneliness (UKB data field 2020-0.0), and risk-taking (UKB data field 2040-0.0).

#### Power calculation

We conducted a power analysis (adapted from www.mv.helsinki.fi/home/mjxpirin/GWAS_course/material/GWAS3.html) to assess the sensitivity of the study design for *SSH2* investigations. Assuming a significance level of α = 0.05/12 = 0.0042 and Minor Allele Frequency (MAF) for *SSH2* of 0.00005, the available sample size (*N* = 423,887) would be sufficient to yield 80% power to detect an effect size of β = 0.5766 (fig. S4). To ensure a conservative estimate, we included the full set of traits, continuous and categorical. Given the likely correlations among traits and use of matched controls, the effective power may in fact be higher than this baseline estimate.

#### TKTL1

Given the profound effects of the derived allele of *TKTL1* on brain development in experiments described by Pinson *et al.* (*10*), we also included targeted investigations of genotype/phenotype relationships for this aSNV. As that prior study had highlighted increased neuronal counts specifically in the frontal lobe (*10*), we focused our phenotypic analysis first on relevant neuroanatomical data, making use of imaging-derived phenotypes generated by an imaging-processing pipeline developed and run on behalf of the UKB (*17*, *59*). Imaging-derived structural measures (UKB category 192) were available for five unrelated carriers (one female; mean age ± SD: 71.2 ± 6.14 years). To estimate total frontal lobe surface area, we summed for each brain hemisphere the following imaging-derived phenotypes superior frontal (UKB data field 26748-2.0 and 26849-2.0), rostral middle frontal (UKB data field 26747-2.0 and 26848-2.0), caudal middle frontal (UKB data field 26724-2.0 and 26825-2.0), pars opercularis (UKB data field 26738-2.0 and 26839-2.0), pars triangularis (UKB data field 26740-2.0 and 26841-2.0), pars orbitalis (UKB data field 26739-2.0 and 26840), lateral orbifrontal (UKB data field 26732-2.0 26833-2.0), medial orbifrontal (UKB data field 26734-2.0 and 26835-2.0), precentral (UKB data field 26744-2.0 and 26845-2.0), paracentral (UKB data field 26737-2.0 and 26838-2.0), and frontal pole (UKB data field 26752-2.0 and 26853-2.0). For the same cortical parcellations, we used the imaging-derived cortical thickness (UKB data field 26782-2.0 and 26883-2.0, UKB data field 26781-2.0 and 26882-2.0, UKB data field 26758-2.0 and 26859-2.0, UKB data field 26772-2.0 and 26873-2.0, UKB data field 76774-2.0 and 26875-2.0, UKB data field 26773-2.0 and 26874-2.0, UKB data field 26766-2.0 and 26867-2.0, UKB data field 26768-2.0 and 26869-2.0, UKB data field 26778-2.0 and 26879-2.0, UKB data field 26771-2.0 and 26872-2.0, UKB data field 26786-2.0 and 26887-2.0) to estimate the averaged cortical thickness for each hemisphere in the frontal lobe. As Pinson *et al.* (*10*) and others (*33*) have suggested that the change from archaic to human *TKTL1* could have played an important role for the evolution of complex behavior, we also looked at qualification level (UKB field 6138-0.0). For each individual, we included only the highest qualification level reported in the analysis.

As *TKTL1* carriers were found in more than one ancestry cluster, the matched samples of noncarriers were set up as follows:

1) For brain imaging phenotypes, we first used all five carriers with imaging data and identified an equal number of individuals homozygous for the derived allele (*N* = 429) per carrier, which were only matched by age (UKB field 21003-2.0) and sex (UKB data field 31-0.0). This resulted in a noncarrier sample across ancestries of 2145 individuals (429 females; mean age ± SD: 71.2 ± 5.49 years).

2) For a sensitivity analysis of the above, we only used the three European carriers (one female; mean age ± SD: 73.67 ± 3.21 years), where 10 noncarrier individuals were matched to each carrier by the first two genetic principal components (PC1 and PC2 ± 2.5, respectively; UKB data field 22009-0.1 and 22009-0.2), age (± 2.5 years; UKB field 21003-2.0), and sex (UKB data field 31-0.0). This resulted in 30 unique noncarriers (10 females; mean age ± SD: 73.2 ± 3.21 years).

3) For our analysis of qualification level, we only selected unrelated carriers, where we could identify 20 unique, matched (PC1 and PC2 ± 2.5, respectively; age ± 2.5 years, sex) noncarriers, which led to a final sample composed of 30 carriers (21 females; 11 AFR, 10 EUR, and 9 uncategorized; mean age ± SD: 54.4 ± 7.67 years) and 600 matched noncarriers (420 females; mean age ± SD: 54.49 ± 7.73 years).

For all included qualitative traits used in this study, we combined possible answer options “Do not know,” “Do not want to answer,” and/or “None of the above” to one item. For both *SSH2* and *TKTL1*, the 95% binomial confidence intervals for categorical traits were computed with the statsmodels package (*60*) in Python using the Wilson method (*61*).
