# Supplementary material for: Evaluating the effects of archaic protein-altering variants in living human adults
Source: Sci Adv. 2025 Dec 10;11(50):eads5703. doi: 10.1126/sciadv.ads5703 (PMC12693971; doi:10.1126/sciadv.ads5703)
Supplement: Supplementary file 1 — Figs. S1 to S4 Legends for tables S1 to S4 [file sciadv.ads5703_sm.pdf]

Science Advances
AAAS

Supplementary Materials for

Evaluating the effects of archaic protein-altering variants in living human adults

Barbara Molz *et al.*

Corresponding author: Simon E. Fisher, simon.fisher@mpi.nl

Sci. Adv. **11**, eads5703 (2025)
DOI: 10.1126/sciadv.ads5703

The PDF file includes:

Figs. S1 to S4
Legends for tables S1 to S4

Other Supplementary Material for this manuscript includes the following:

Tables S1 to S4

Fig. S1.

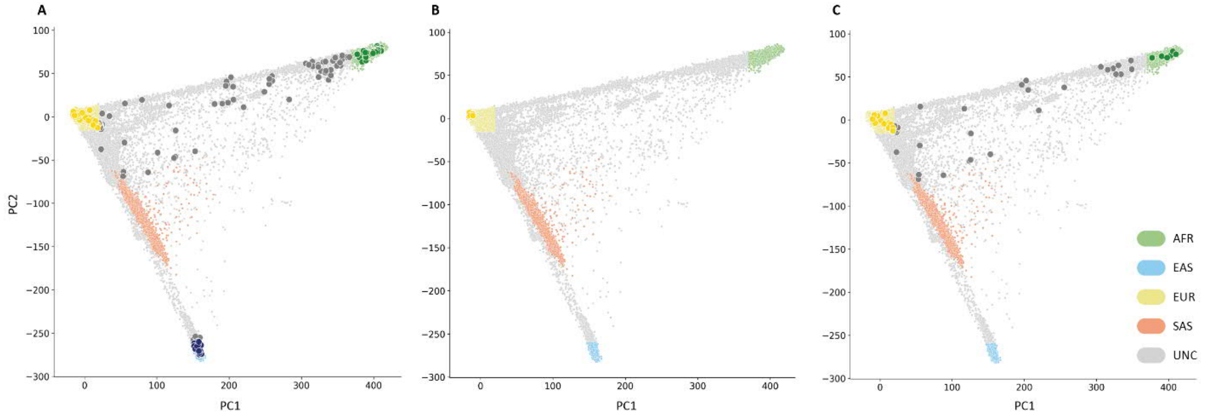

Fig. S1. Ancestry distribution of identified aSNV carriers. Top two principal components plotted for (**A**) all aSNV carriers (N = 165), (**B**) carriers of an aSNV in *SSH2* (N = 21), and (**C**) carriers of an aSNV in *TKTL1* (N = 62). Carriers are overlaid as darker shaded dots over individuals that do not carry an aSNV. Ancestry superclusters are colour coded and were defined based on the top 4 principal components using self-reported background (UKB data field 21000); AFR = African, EAS = East Asian, EUR = European, SAS = South Asian, UNC = Uncategorized.

Fig. S2.

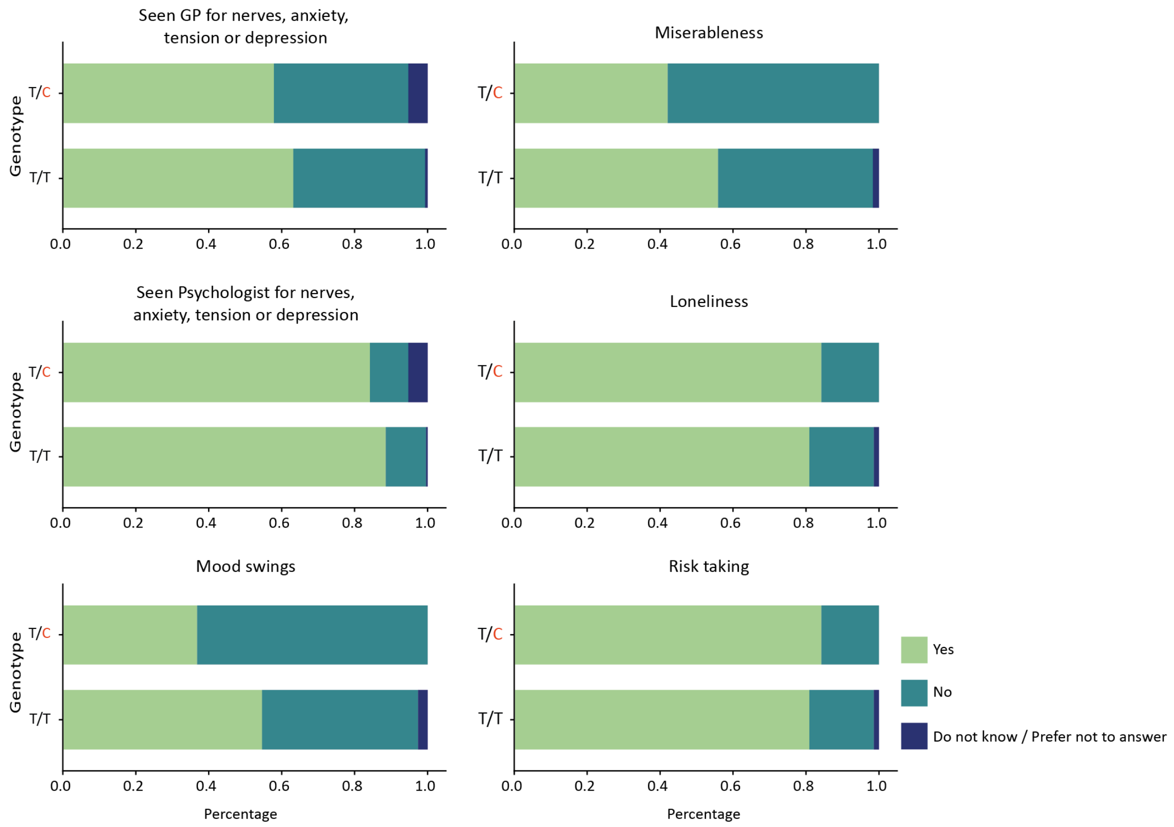

Fig. S2. Neuropsychiatric traits in carriers of an archaic SNV in *SSH2*. Stacked bar plots highlight the percentage of answers (Yes, No, Do not know/Prefer not to answer) with respect to a set of categorical traits within the neuropsychiatry domain per genotype (N_{aSNV} = 19; $N_{Non\text{-}carrier}$ = 39,501). See Table S2 for trait percentages with 95% binomial confidence intervals.

Fig. S3.

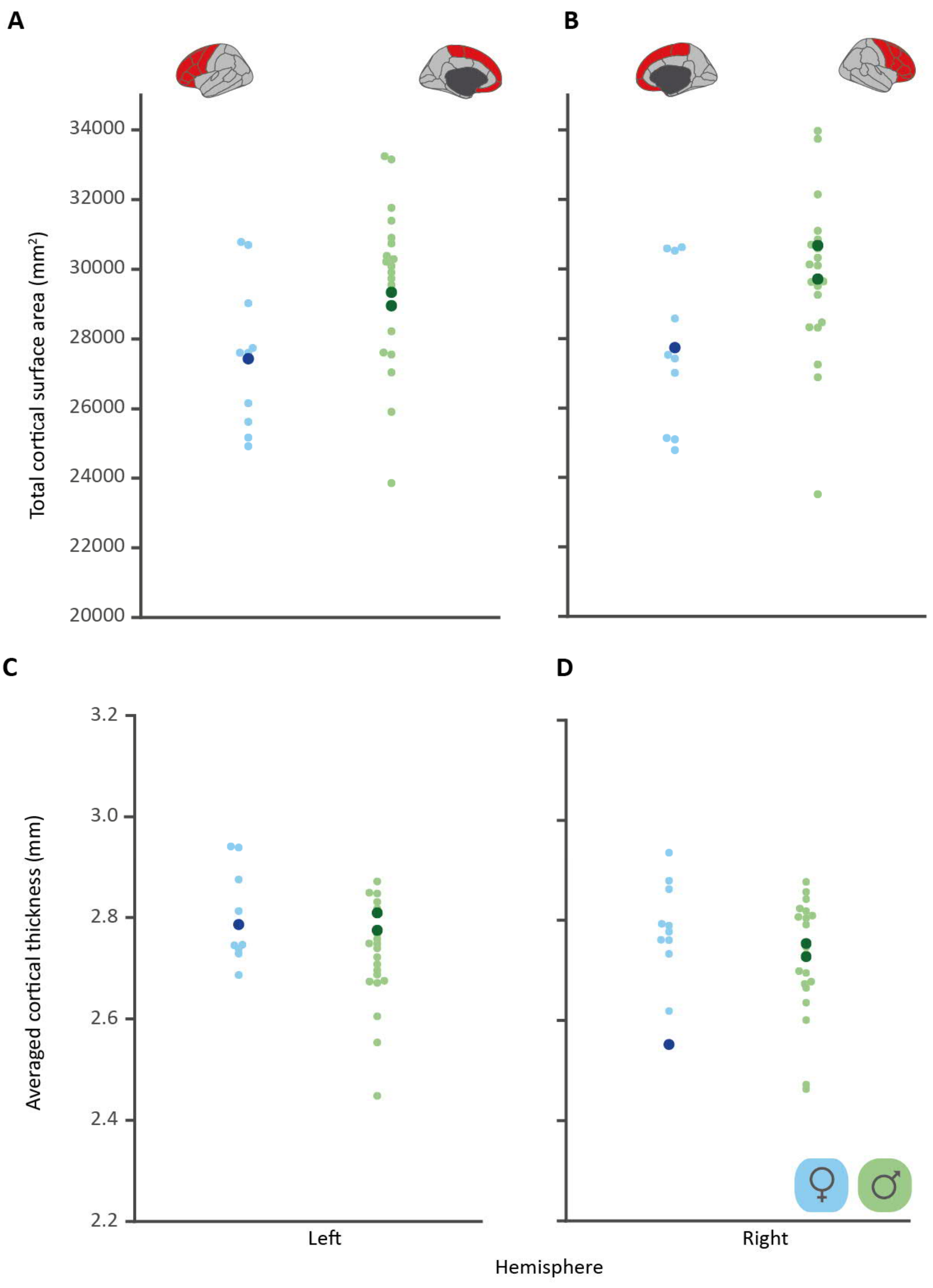

Fig. S3. **Carriers of the archaic allele of the *TKTL1* aSNV show no diverging cortical measures compared to a matched set of non-carriers.** Strip plots depict the phenotypic variability in both female (blue) and male (green) matched samples of non-carriers for total frontal lobe cortical surface area for both left and right hemisphere (**A** and **B**, respectively) and averaged frontal lobe cortical thickness (**C** and **D**, respectively). Carrier values for each sex and metric are overlaid as darker shaded dots. In this sensitivity analysis, only participants with European ancestry were included ($N_{aSNV} = 3$, $N_{Non\text{-}carrier} = 30$).

Fig. S4.

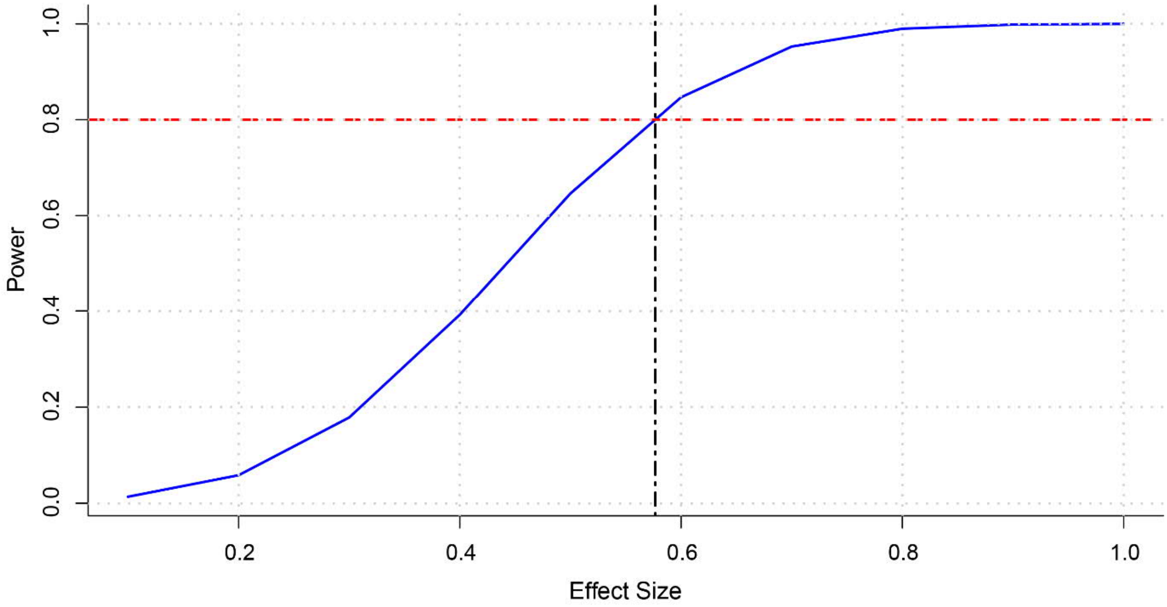

Fig. S4. Power curve illustrating the relationship between effect size and statistical power. Power curve across a range of effect sizes for the queried position on chr17:29632016 within *SSH2*. Total sample size and aSNV allele frequency (see Table 1) as well as an adjusted alpha level of 0.0042 was used in the calculations. The red horizontal dashed line marks the 80% power threshold, while the dashed vertical line indicates the corresponding minimum effect size (0.5766) needed to reach this level.

Table S1. < in a separate .xlsx file >
Trait Percentages with 95% Binomial Confidence Intervals
Percentages and 95% confidence intervals (CI) per genotype for all categorical traits shown in Figure 1.

Table S2. < in a separate .xlsx file >
Trait Percentages with 95% Binomial Confidence Intervals
Percentages and 95% confidence intervals (CI) per genotype for all categorical traits shown in Figure S2.

Table S3. < in a separate .xlsx file >
Trait Percentages with 95% Binomial Confidence Intervals
Percentages and 95% confidence intervals (CI) per genotype for all categorical traits shown in Figure 3.

Table S4. < in a separate .xlsx file >
Overview of all genomic positions of interest. Based on the Kuhlwilm & Boeckx (3) catalogue of single nucleotide changes that distinguish modern humans and archaic hominins, we include all putative fixed genomic locations with an allele frequency of one (AF = 1) at the time of publication of that prior study, as well as the high-frequency change on *TKTL1*. Please note, rsIDs refer to the change from derived to archaic allele while amino-acid changes indicate the change that occurred after *Homo sapiens* split from its common ancestor with Neanderthals (archaic_position_derived); *excluded position as main transcript was in an intron; **excluded as position did not pass variant quality control; ***excluded as position/change could not be unambiguously identified.
